# Psl Produced by Mucoid *Pseudomonas aeruginosa* Contributes to the Establishment of Biofilms and Immune Evasion

**DOI:** 10.1128/mBio.00864-17

**Published:** 2017-06-20

**Authors:** Christopher J. Jones, Daniel J. Wozniak

**Affiliations:** a Center for Microbial Interface Biology and Department of Microbial Infection and Immunity, Ohio State University, Columbus, Ohio, USA; b Department of Internal Medicine, Division of Pulmonary, Critical Care, & Sleep Medicine, Ohio State University, Columbus, Ohio, USA; c Department of Microbiology, Ohio State University, Columbus, Ohio, USA; Emory University School of Medicine

**Keywords:** biofilms, *Pseudomonas aeruginosa*, cystic fibrosis, exopolysaccharide, immune evasion, lung infection

## Abstract

Despite years of research and clinical advances, chronic pulmonary infections with mucoid *Pseudomonas aeruginosa* remain the primary concern for cystic fibrosis patients. Much of the research on these strains has focused on the contributions of the polysaccharide alginate; however, it is becoming evident that the neutral polysaccharide Psl also contributes to biofilm formation and the maintenance of chronic infections. Here, we demonstrate that Psl produced by mucoid strains has significant roles in biofilm structure and evasion of immune effectors. Though mucoid strains produce less Psl than nonmucoid strains, the Psl that is produced is functional, since it mediates adhesion to human airway cells and epithelial cell death. Additionally, Psl protects mucoid bacteria from opsonization and killing by complement components in human serum. Psl production by mucoid strains stimulates a proinflammatory response in the murine lung, leading to reduced colonization. To determine the relevance of these data to clinical infections, we tested Psl production and biofilm formation of a panel of mucoid clinical isolates. We demonstrated three classes of mucoid isolates, those that produce Psl and form robust biofilms, those that did not produce Psl and have a poor biofilm phenotype, and exopolysaccharide (EPS) redundant strains. Collectively, these experimental results demonstrate that Psl contributes to the biofilm formation and immune evasion of many mucoid strains. This is a novel role for Psl in the establishment and maintenance of chronic pulmonary infections by mucoid strains.

## IMPORTANCE

Cystic fibrosis patients are engaged in an ongoing battle against chronic lung infections by the bacterium *Pseudomonas aeruginosa*. One key factor contributing to the maintenance of chronic infections is the conversion to a mucoid phenotype, where the bacteria produce copious amounts of the polysaccharide alginate. Once the bacteria become mucoid, existing treatments are poorly effective. We proposed that mucoid bacteria produce an additional polysaccharide, Psl, which is important for their establishment and maintenance of chronic infections. This work demonstrates that Psl enhances attachment of mucoid bacteria to lung surfaces and leads to inflammation and damage in the lung. Additionally, we find that 50% of mucoid bacteria isolated from patients with chronic infections rely on Psl for the structure of their biofilm communities, suggesting that treatments against Psl should be investigated to enhance the success of current therapies.

## INTRODUCTION

The Gram-negative pathogen *Pseudomonas aeruginosa* is a significant burden on the health care industry, with up to 10% of nosocomial infections attributed to this pathogen (1, 2). These infections can present as acute and chronic infections of burn wounds, skin, and indwelling medical devices and can disseminate, resulting in sepsis (1, 2). However, chronic pulmonary infections caused by *P. aeruginosa* are the most prevalent threat to the health and well-being of the nation’s 30,000 cystic fibrosis (CF) patients, with more than 80% of adult patients harboring these infections (3). Patients are initially infected with nonmucoid environmental strains of *P. aeruginosa*; however, over time, mutations in *mucA*, encoding an anti-sigma factor result in the overproduction of the polysaccharide alginate, termed mucoidy (4). Isolation of mucoid strains from sputum is associated with the transition from intermittent to chronic infection and poor outcomes (5, 6). In addition to the underlying disease and direct tissue damage caused by the bacteria, there is a robust, though ineffective immune response that exacerbates the disease (7, 8). Together, these factors cause the reduced lung function and life span associated with CF.

Of the many virulence factors produced by *P. aeruginosa* that exacerbate disease, the three polysaccharides alginate, Psl, and Pel are the most relevant with regard to the establishment of chronic biofilm infections and immune evasion (9, 10). Each of these polysaccharides contributes to the properties of the collective biofilm. Alginate is a negatively charged hygroscopic acetylated polymer with nonrepetitive monomers of β-1,4-linked l-guluronic and d-mannuronic acids (11). The biosynthetic genes are encoded in an operon that begins with *algD* (5). This operon is tightly regulated, as production of alginate requires a significant energetic commitment through the use of sugar-nucleotide precursors in its production. The *algD* operon is in the regulon of the AlgT (also referred to as AlgU [6] and sigmaE [12]) sigma factor (13). In nonmucoid strains, this sigma factor is sequestered at the cell membrane and rendered inactive by the anti-sigma factor MucA. When MucA is truncated by a mutation, it is no longer able to interact with AlgT. This allows AlgT to bind to the *algD* promoter, and overexpression of alginate occurs. Alginate contributes to chronic disease by inhibiting phagocytosis and bacterial damage by reactive oxygen species (ROS) (14–16).

Psl is a neutral branched pentasaccharide containing d-mannose, d-glucose, and l-rhamnose in a 3:1:1 ratio (17). This polysaccharide plays a role in biofilm structure and interactions with surfaces (17–20). There are two forms of Psl: a high-molecular-weight cell-associated component and a relatively smaller soluble form of Psl that can be isolated from cell-free culture supernatant. Psl prevents complement activation and opsonization, hindering bacterial killing by phagocytes (21, 22). Additionally, Psl mediates attachment to lung epithelial cells. Psl expression elicits a proinflammatory response from epithelial cells by indirectly activating the NF-κB cascade, perpetuating the damage caused by infection and inflammation (23).

Pel is a positively charged polysaccharide composed of partially acetylated 1→4 glycosidic linkages of *N*-acetylgalactosamine and *N*-acetylglucosamine (24). Pel was initially identified, and subsequently named, based on its role in pellicle formation on liquid cultures (18, 25). Other functions attributed to Pel include maintaining cell-cell interactions and antibiotic tolerance (26, 27) and facilitating surface attachment (28). A recent report indicates that Pel stabilizes the biofilm matrix by cross-linking extracellular DNA (eDNA) to the biofilm stalk via ionic interactions (24). Taken together, it is clear that Pel can play a significant role in the biofilm matrix.

Clinical and environmental isolates demonstrate different requirements for each of the three polysaccharides during biofilm formation. Colvin et al. divide clinical isolates into four classes based on the polysaccharide requirement (29). Their study revealed that Psl and Pel are functionally redundant in the biofilm matrix for many strains of *P. aeruginosa*; however, some strains require one polysaccharide or the other for biofilm formation. All of these analyses were performed in nonmucoid strains; however, there is evidence that Psl is important for biofilms and infections caused by mucoid strains. For example, Psl is expressed by mucoid strains, and in murine models of infection, treatment with antibodies against Psl aids clearance of infections caused by both mucoid and nonmucoid *P. aeruginosa* strains (30–33). Here, we demonstrate that the Psl produced by mucoid strains is a significant modulator of immune interaction and colonization, with roles in biofilm structure, pulmonary colonization, and immune evasion. We propose that the development of methodologies for blocking or degrading Psl would improve treatment regimens and outcomes for patients harboring chronic infections by mucoid strains.

## RESULTS AND DISCUSSION

### Psl contributes to the mucoid biofilm matrix.

Previous research on the contributions of Psl to the biofilm matrix have focused on its role in nonmucoid strains. Though mucoid strains produce less Psl on average than nonmucoid strains do (31, 32), there is evidence that Psl has a function in the matrix of mucoid bacteria (30, 33). Deletion of the *psl* operon promoter (*P*. *aeruginosa* strain PDO310 [no production of Psl {Psl−}]) or introduction of an inducible promoter controlling expression of the *psl* operon (strain PDO320 [high production of Psl {Psl+++}]) allows modulation of Psl production in the mucoid parental strain PDO300 (weak production of Psl [Psl+]). Isogenic nonmucoid *P*. *aeruginosa* PAO1 derivatives (WFPA800 [Psl−] and WFPA801 [Psl+++]) were also used in this study (see Fig. S1 and Table S1 in the supplemental material).

10.1128/mBio.00864-17.2 FIG S1  Quantification of polysaccharide production. (A) Psl was isolated from stationary-phase cultures and quantified by immunoblotting. Three biological replicates were performed in triplicate. The average Psl concentration plus standard error of the mean (SEM) is depicted. (B) Alginate was isolated from cultures grown on plates and quantified by carbazole assay. Three biological replicates were performed in triplicate. The average alginate concentration ± standard error of the mean is depicted. These assays were utilized to characterize the polysaccharide production (−, no production; +/++, weak or intermediate production; +++, high production) in order to simplify strain designations. These polysaccharide production keys are utilized throughout the figures. Download FIG S1, TIF file, 0.1 MB. Copyright © 2017 Jones and Wozniak. 2017 Jones and Wozniak This content is distributed under the terms of the Creative Commons Attribution 4.0 International license.

10.1128/mBio.00864-17.4 TABLE S1 Strains used in this study. Download TABLE S1, DOCX file, 0.1 MB. Copyright © 2017 Jones and Wozniak. 2017 Jones and Wozniak This content is distributed under the terms of the Creative Commons Attribution 4.0 International license.

**FIG 1:**
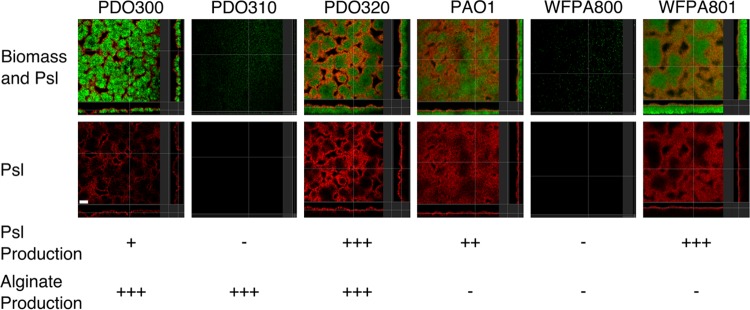
Psl contributes to the biofilm matrix of mucoid strains. (A) Green fluorescent protein (GFP)-expressing biofilms (green) were grown for 48 h and stained with an anti-Psl cocktail (red). Orthogonal images obtained with confocal microscopy determine the localization and contribution of Psl in the matrix. The following strains were tested: PDO300 (mucoid, Psl+), PDO310 (mucoid, Psl−) PDO320 (mucoid, Psl+++), PAO1 (nonmucoid, Psl++), WFPA800 (Psl−), and WFPA801 (nonmucoid, Psl+++). Three biofilms were imaged in duplicate. Bar, 30 µm. The Psl and alginate production of each strain has been indicated below the images. Polysaccharide quantification is shown in Fig. S1 in the supplemental material.

To determine the expression level and localization of Psl in biofilms formed by mucoid and nonmucoid strains, 2-day-old flow cell biofilms were stained with anti-Psl monoclonal antibodies (33) and imaged by confocal laser scanning microscopy (CLSM) (Fig. 1). In all strains capable of producing Psl, the anti-Psl antibodies bound at the surface of the biofilm, furthest away from the substrate. This agrees with previous observations, which described lectin-stained Psl as a “shell” around the biofilm (34). Strains lacking the *psl* promoter exhibited no staining with the anti-Psl antibody. It is important to note that there is less anti-Psl staining material in the mucoid strain than in the nonmucoid strain, though the pattern of Psl localization is similar. Previous studies have indicated that mucoid strains produce approximately 50 to 60% of the amount of Psl produced by the nonmucoid isogenic strains (31, 32). It is reasonable to predict that from the similarities in Psl localization in these images that the Psl produced by mucoid biofilms performs similar structural functions. We next investigated the functional role of Psl in the mucoid biofilm.

**FIG 2:**
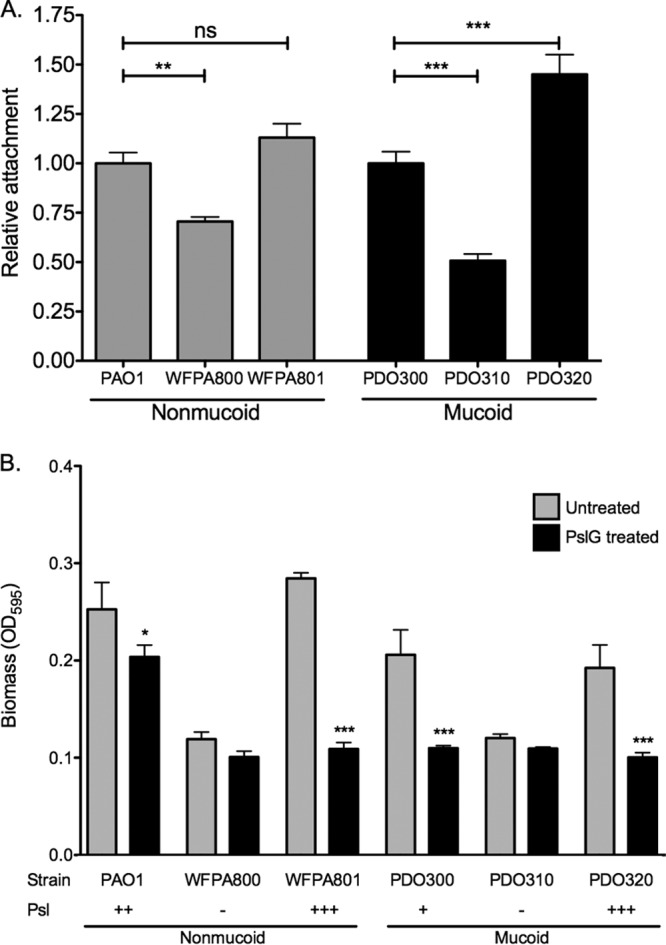
Psl is a structural component of the mucoid biofilm matrix. (A) Four-hour peg biofilms were grown with the indicated strains, and biomass was stained with crystal violet. Results were normalized to the value for the isogenic parental strain. Three biological replicates were performed in triplicate. Statistical significance was determined by one-way ANOVA, followed by Dunnett’s multiple-comparison test (**, *P* ≤ 0.01; ***, *P* ≤ 0.001; ns, not significant). (B) PslG treatment reduces adherent mucoid biomass. The Psl hydrolase PslG (86 nM) was added to biofilms to determine the structural contribution of Psl to the biofilm matrix. Crystal violet staining revealed adherent biomass after treatment. Three biological replicates were treated in triplicate. The values for untreated samples were compared to the values for treated samples by a one-way ANOVA, followed by Bonferroni’s posthoc test (*, *P* ≤ 0.05; ***, *P* ≤ 0.001).

Image analysis software COMSTAT of 48-h biofilms and peg biofilm assays reveal that both mucoid and nonmucoid biofilms require Psl for surface attachment and development of biomass, as the Psl-null strains in mucoid (PDO310) or nonmucoid (WFPA800) backgrounds are unable to form biofilms reminiscent of the isogenic parent strain (Fig. 2A and Fig. S2). The Psl-overexpressing strain forms biofilms with increased thickness and biomass, further emphasizing the structural contribution of Psl to mucoid biofilms. To investigate whether the increased biomass formed by the Psl-producing strains was due to the production of Psl rather than other targets which are coregulated with Psl, we treated preformed biofilms with exogenous PslG hydrolase (Fig. 2B). Addition of the Psl hydrolase significantly reduces the biofilm biomass in all Psl-producing strains tested. In the mucoid strains PDO300 and PDO320, the biomass is nearly eradicated to background levels, as indicated by the Psl mutant PDO310. This highlights the importance of Psl to the structure of mucoid biofilms. Treatment of nonmucoid biofilms with the hydrolase significantly reduces the biomass; however, PAO1 is not reduced to Psl-null biomass levels. This could indicate a role for the Pel polysaccharide, which contributes to the PAO1 biofilm structure. Since Psl and Pel share a precursor, overexpression of one reduces the expression of the other (31). Therefore, the difference between the PslG-treated biofilms by strains PAO1 and WFPA801 is likely due to Pel produced by PAO1, but not WFPA801.

10.1128/mBio.00864-17.3 FIG S2  COMSTAT analysis of biofilm images. Quantitative data were obtained with the COMSTAT2 software package, followed by a one-way ANOVA with Dunnett’s multiple-comparison test (**, *P* ≤ 0.01; ***, *P* ≤ 0.001). Nine images were analyzed per condition. Download FIG S2, TIF file, 0.1 MB. Copyright © 2017 Jones and Wozniak. 2017 Jones and Wozniak This content is distributed under the terms of the Creative Commons Attribution 4.0 International license.

### Psl promotes interactions between mucoid strains and host cells.

**FIG 3:**
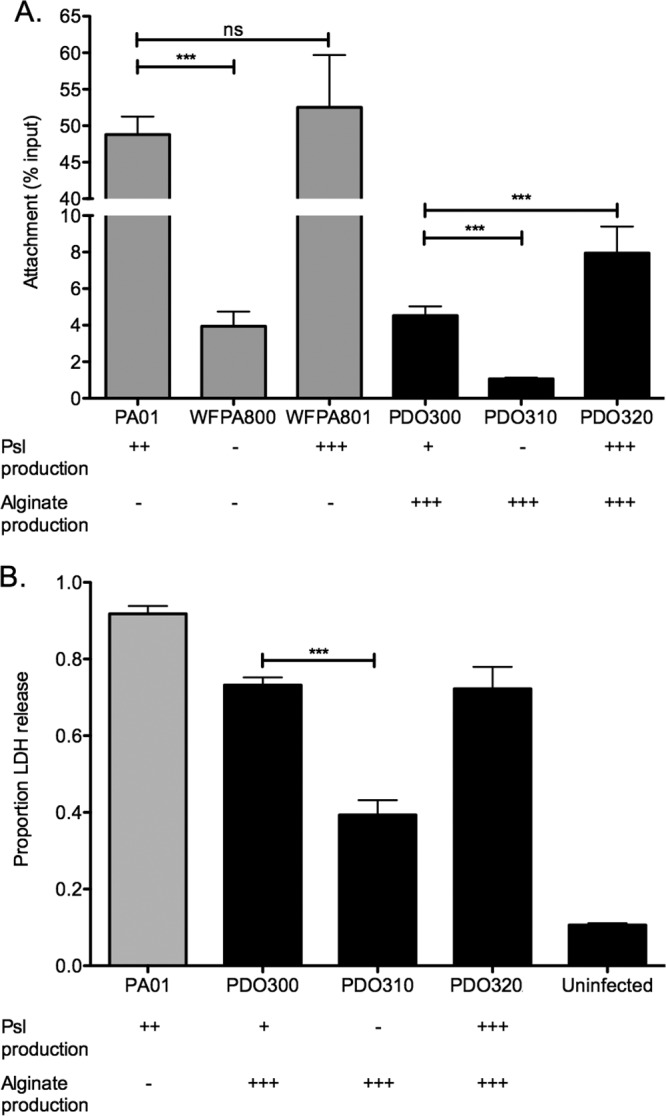
Psl significantly increases attachment to lung epithelial cells by both mucoid and nonmucoid strains. (A) Confluent A549 lung epithelial cells were infected with bacterial cells at an MOI of 10 for 1 h. After the cells were washed, bacteria that adhered to the epithelial cells were enumerated (CFU). The percent attachment was determined by comparing the CFU recovered to the CFU of the infection dose. Two biological replicates were performed in triplicate. Statistical significance was determined by one-way ANOVA, followed by Dunnett’s multiple-comparison test (***, *P* ≤ 0.001; ns, not significant). Each mutant was compared to its respective mucoid or nonmucoid parental strain. (B) Psl enhances cytotoxicity of lung epithelial cells. Confluent A549 lung epithelial cells were infected with bacterial cells at an MOI of 10 for 16 h. Release of lactate dehydrogenase (LDH) was measured using the CytoTox 96 nonradioactive cytotoxicity assay. Five independent experiments were performed in triplicate. Statistical significance was determined by a one-way ANOVA, followed by Dunnett’s multiple-comparison test (***, *P* ≤ 0.001). Each mutant was compared to the mucoid parental strain PDO300.

In nonmucoid cells, Psl promotes attachment to epithelial cells *in vitro*, contributing to host cell killing and immune response (23). Since we demonstrated that Psl plays a similar structural role in mucoid and nonmucoid biofilms, we next investigated whether Psl also mediates interactions between mucoid *P. aeruginosa* and host cells. Monolayers of A549 lung epithelial cells were infected with exponential-phase cultures of *P. aeruginosa* at a multiplicity of infection (MOI) of 10. Following 1 h of incubation and washing, samples were homogenized, and the number of CFU of adherent bacteria was compared to the number of CFU of the infection dose to determine the percentage attached to the monolayer (Fig. 3A). Strain PDO300 attaches at about 10% the rate of the nonmucoid strain PAO1 (4.5% and 48%, respectively). This is expected considering the reduced production of Psl in mucoid strains and previous research identifying Psl as an adhesin in nonmucoid strains (23). Although attachment is reduced in the mucoid strain, deletion of the *psl* promoter significantly reduces attachment of the mucoid strain (1.1%). The attachment phenotype is rescued in the Psl overexpression strain (8.0%). Collectively, these data indicate that though the adhesion of mucoid cells to human epithelial cell lines is reduced compared to nonmucoid strains, this interaction is mediated through Psl.

### Psl enhances cytotoxicity by mucoid strains.

We next wished to determine how Psl-mediated attachment to lung epithelial cells affects the viability of epithelium. Monolayers of A549 cells were infected for 1 h as described above, followed by replacement with fresh sterile medium. After a 16-h incubation, cell culture supernatants were tested for lactate dehydrogenase (LDH) activity (Fig. 3B). LDH is an intracellular enzyme that is released into the supernatant from eukaryotic cells upon death. This enzyme can be used as a proxy measurement for cell death. As a control for total cell lysis, positive-control samples were incubated with lysis detergent for 45 min prior to collection of supernatants. Percent LDH release is reported as the percentage of LDH activity of the test sample compared to that of the detergent-lysed cells. Infection with nonmucoid strain PAO1 resulted in 91.8% of the LDH activity of the control cell population, while infection with the mucoid strain PDO300 resulted in lysis of 73.0% of the A549 cells. The reduced killing by PDO300 is most likely due to AlgR-dependent downregulation of the type three secretion system (T3SS) in mucoid strains (35, 36), though it is possible that the loss of T3SS observed in mucoid strains is also a pathoadaptation to chronic infection in the lung (37). Since PDO300 is a lab-derived mucoid strain that has never been exposed to the selective pressure of the lung, it may produce more T3SS effectors than clinically isolated mucoid strains, accounting for the relatively high cytotoxicity induced by infection with this strain (37). The Psl− strain PDO310 was significantly less cytotoxic than PDO300, only lysing 39.3% of cells. The cytotoxicity is restored when Psl is induced in PDO320 (72.3%). In a uninfected sample, only 10.3% of the cells were lysed, suggesting that the cell lysis observed was due to the infection and not culture conditions. These data suggest that Psl-mediated cell adhesion facilitates cytotoxicity of epithelial monolayers by mucoid strains.

### Opsonization of mucoid strains is inhibited by Psl.

**FIG 4:**
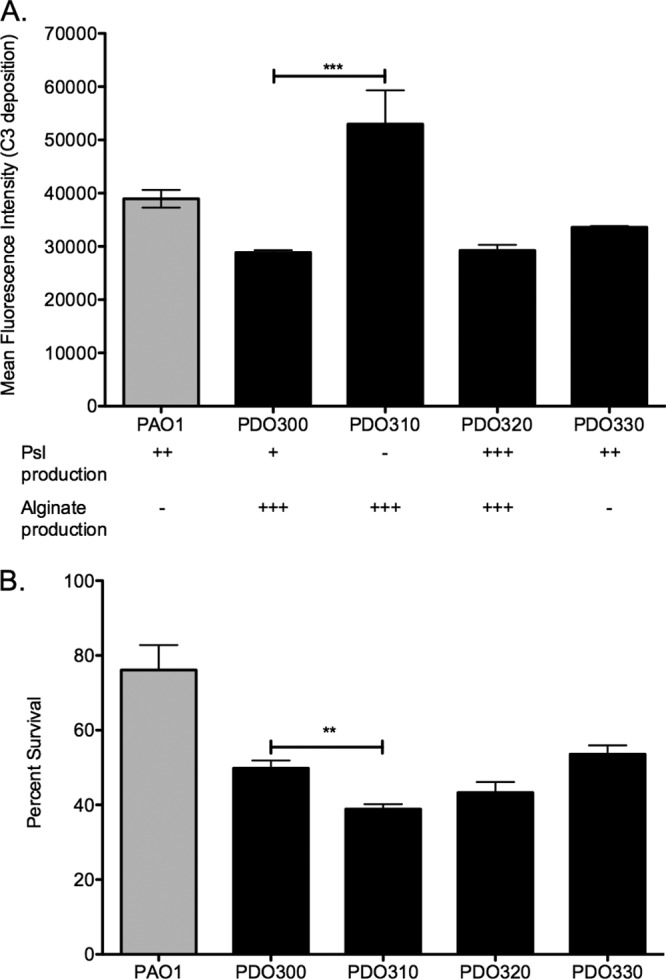
Psl protects mucoid strains from serum components. (A) Psl production inhibits deposition of complement C3 on mucoid strains. Exponential-phase cells were incubated with 20% normal human serum for 5 min, then stained with an antibody against human complement C3, and analyzed for C3 deposition by flow cytometry. Six biological replicates were performed in triplicate. Statistical significance was determined by a one-way ANOVA, followed by Dunnett’s multiple-comparison test (***, *P* ≤ 0.001). Each mutant was compared to the mucoid parental strain PDO300. (B) Psl production protects mucoid bacteria from killing by serum. Bacterial suspensions were treated with human serum for 20 min and plated to determine bacterial survival. Data are reported as the percentage of serum-treated bacteria recovered compared to PBS-treated samples. Three experiments were performed in triplicate, and statistical significance was determined by one-way ANOVA, followed by Dunnett’s multiple-comparison test (**, *P* ≤ 0.01).

Since Psl mediates increased inflammation by epithelial cells, we next wanted to determine the role of Psl produced by mucoid strains in the interaction with the innate immune system. Mishra et al. previously reported that in nonmucoid *P. aeruginosa*, Psl blocks deposition of the complement component C3 (21). We reasoned that this immune evasive effect might be duplicated in mucoid strains, despite the coating of alginate potentially masking the surface-associated Psl from serum components and phagocytes. To test this, bacteria were incubated with pooled normal human serum, followed by washing and staining for deposition of the complement component C3. Flow cytometry determined the amount of C3 antibody bound to each bacterial cell, reported as the mean fluorescence intensity (MFI) for each sample (Fig. 4A). Deletion of the *psl* promoter from the mucoid strain PDO300 resulted in a significant increase in the amount of C3 deposited on the surface of the bacterium. This effect could be complemented by inducing Psl production in PDO320. To demonstrate that this effect was due to Psl produced by the mucoid strain and not an artifact from alginate overexpression, C3 deposition was determined on the Δ*algD* mutant strain PDO330. This mutation renders the parental PDO300 nonmucoid but leaves the AlgT regulatory activity intact. C3 deposition on PDO330 was indistinguishable from PDO300, suggesting that Psl, not alginate, inhibits the C3 deposition on mucoid strains.

### Psl production by mucoid strains enhances serum resistance.

To further investigate the protective role of Psl against human serum components, we performed a serum killing assay. Bacteria were incubated with 50% human serum for 20 min and then plated to determine resistance to serum-mediated killing (Fig. 4B). The PAO1 strain was significantly more resistant to serum killing than its mucoid derivative PDO300, which is a common phenotype of mucoid strains that is associated with high-molecular-weight O-antigen-deficient LPS in these strains (7, 31, 38, 39). The Psl-null mutant PDO310 was significantly more susceptible to killing by serum than the Psl-proficient parental strain, which is expected from the increased C3 deposition on this strain shown in Fig. 4A. Complementation of Psl production in this strain (PDO320) returns the serum sensitivity to the level of the mucoid parental strain. The serum sensitivity of the alginate-null strain PDO330 was similar to that of the parental PDO300, demonstrating that the decreased serum resistance is specifically due to Psl. Taken together, these results suggest that Psl protects mucoid strains from both opsonization and killing in the presence of serum.

### Psl induces a proinflammatory response and clearance from the murine lung.

**FIG 5:**
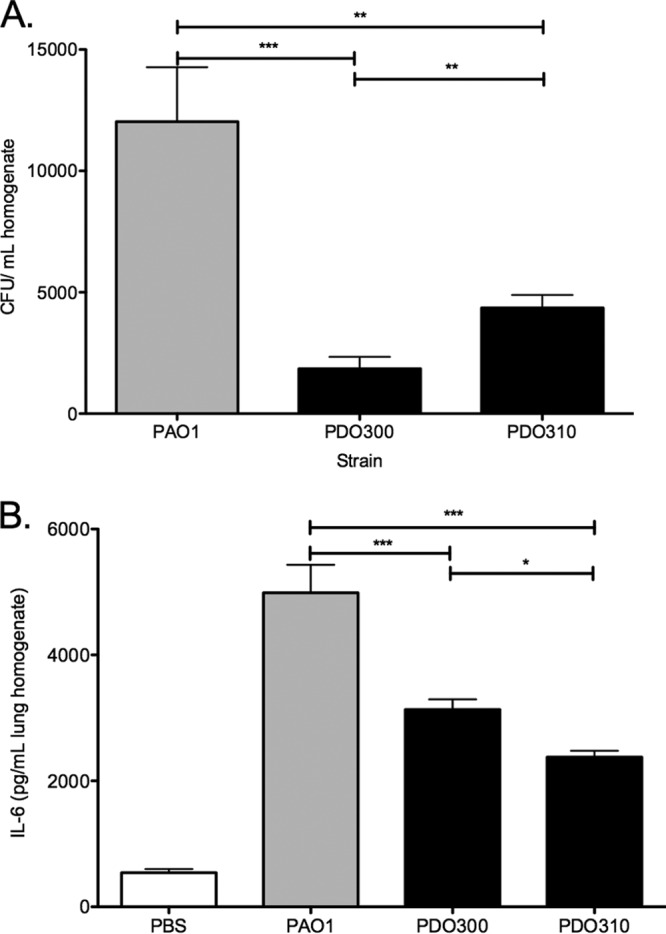
Psl induces a proinflammatory response and clearance from the murine lung. BALB/c mice were infected intranasally, and lungs were harvested and homogenized at 24 h postinfection. (A) Samples were diluted and plated to determine bacterial load. Three homogenates were plated in triplicate for each strain infection. (B) IL-6 was quantified from lung homogenates by ELISA. Three homogenates were tested in triplicate for each strain infection. Statistical significance was determined by one-way ANOVA, followed by Bonferroni’s posthoc test (*, *P* ≤ 0.05; **, *P* ≤ 0.01; ***, *P* ≤ 0.001).

All *in vitro* lines of evidence in this study suggest that Psl plays a significant role for mucoid cells in biofilm formation, initial epithelium attachment, and immune interaction. To investigate whether these phenotypes persist in the more complex setting of an *in vivo* infection, we tested the abilities of several strains to colonize and induce inflammation in an acute murine pulmonary infection model. BALB/c mice were intranasally inoculated with suspensions of bacterial strains. At 24 h postinfection, lungs were aseptically harvested and homogenized. The homogenate was plated to determine pulmonary bacterial load, and interleukin 6 (IL-6) was measured as an indicator of the proinflammatory response (Fig. 5). We hypothesized that the increased attachment to the epithelia would lead to increased colonization. At 24 h postinfection, the nonmucoid strain PAO1 was approximately 20 times more efficient at colonizing the murine lung than its mucoid derivative, PDO300 (Fig. 5A). The mucoid, Psl-null strain PDO310 colonized the lung significantly more efficiently than PDO300 did. This was surprising, as the *in vitro* data indicated that strains capable of producing Psl were able to adhere to lung epithelium more efficiently than Psl-null strains were (Fig. 3).

Byrd et al. reported that A549 cells respond to infection with nonmucoid bacteria by inducing the NF-κB pathway and producing increased amounts of the proinflammatory cytokine IL-8 (23). Further analysis determined that Psl-mediated adhesion to epithelial cells is indirectly responsible for this proinflammatory response. The authors concluded that the Psl production increased the immune response by enhancing the contact between the epithelial cells and the flagellin of *P. aeruginosa*, as the proinflammatory response was absent in a Δ*fliC* mutant, regardless of Psl production. Since we observed Psl-dependent cell adhesion and cytotoxicity by mucoid strains, we investigated whether these strains also induce inflammation. We reasoned that Psl produced by strain PDO300 may induce more inflammation than strain PDO310, leading to enhanced clearance of PDO300. We investigated the inflammatory response in the lung during infection by IL-6 enzyme-linked immunosorbent assay (ELISA) (Fig. 5B), which is an indicator of a strong proinflammatory response at this stage of infection (40, 41). Pulmonary infections with PDO300 induced significantly more IL-6 than those with PDO310 (3,904 versus 2,809 pg/ml, respectively), though more PDO310 was recovered from the lungs (Fig. 5A). Strain PAO1 induced significantly more IL-6 production than either mucoid strain; however, this may simply be due to the 10- to 20-fold increase in bacterial load in the lung. These data suggest that the strong proinflammatory response induced by Psl may lead to more efficient clearance of mucoid strains.

These data expand on a previous report indicating that there is no difference in mortality between mice infected with strains PAO1 and PDO300, with the mice succumbing to infections with either strain between 40 and 50 h (42). That study, however, did not investigate bacterial load or immune response during these infections, which is a key difference between the two studies. Here, we determine that Psl produced by mucoid strains enhances clearance from the lung and IL-6 production at 24 h postinfection. These differences are masked when only mortality is reported, yet they may be important for the maintenance of pulmonary infections by mucoid strains. We suggest that differences in bacterial load and immune response are a more clinically relevant measure of infection severity.

### Mucoid clinical isolates vary in production of Psl.

**FIG 6:**
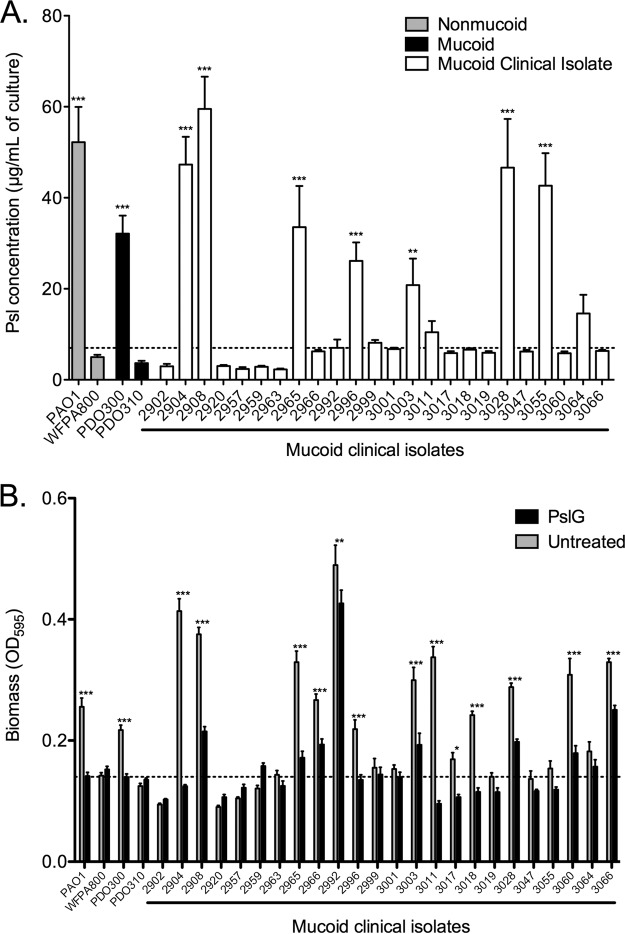
Mucoid clinical isolates display differing levels of production and dependence on Psl. (A) Psl was isolated from stationary-phase cultures and quantified by immunoblotting. Three biological replicates were performed in triplicate. The average Psl concentration is depicted. Statistical significance was determined by a one-way ANOVA, followed by Dunnett’s multiple-comparison test (**, *P* ≤ 0.01; ***, *P* ≤ 0.001). Each mutant was compared to the mucoid P*psl* mutant PDO310. The dotted line depicts double the amount of Psl produced by strain PDO310 for reference. (B) Peg biofilm assays were performed to determine the biofilm-forming potential of mucoid clinical isolates. Biofilms were grown for 4 h, followed by a 1-h treatment with PBS (gray bars) or 86 nM PslG glycosyl hydrolase. Crystal violet staining revealed adherent biomass after treatment. Three biological replicates were treated in triplicate. Statistical significance was determined by a two-way ANOVA, followed by Bonferroni’s posthoc test to compare untreated to PslG-treated samples (*, *P* ≤ 0.05; **, *P* ≤ 0.01; ***, *P* ≤ 0.001).

All of the data in this article were produced using the mucoid derivative of strain PAO1, strain PDO300. This strain background was chosen because it is isogenic to PAO1, and therefore, effects of specific mutations are more easily controlled. To determine whether the roles ascribed to Psl are conserved in mucoid strains, we determined Psl production by 24 mucoid clinical sputum isolates from our strain collection (Fig. 6A). Seven of the 24 isolates produced Psl (defined as significantly more than PDO310). An additional 12 strains produced at least double the amount of Psl as PDO310, but this was not significantly more than PDO310. Five isolates produced the same or smaller amount of Psl as PDO310 did. This result was not surprising, as clinical isolates are genetically diverse. Additionally, these data support the four classes of polysaccharide requirements initially proposed by Colvin (29). On the basis of these classifications, PDO300 is a class II, or Psl-dominant biofilm matrix strain. Colvin et al. had identified PAO1 as a class II strain; however, our data indicate that this classification holds even after mucoid conversion alters the composition of the biofilm matrix. The Psl production data in Fig. 6A suggest that at least 7 of the clinical isolates are also class II strains that rely exclusively on Psl, while the remaining 17 isolates are likely class I (Pel-dominant matrix) or class III (EPS redundant matrix). In order to further define the role of Psl in the biofilm matrix and confirm this classification, 4-h peg biofilm assays were grown, and the adherent biomass was quantified with crystal violet (Fig. 6B, gray bars). Treatment of biofilms with the glycosyl hydrolase PslG determines the structural role of Psl in the matrix of each strain (Fig. 6B). These assays confirm that seven of the strains are class II (strains 2904, 2908, 2965, 2996, 3003, 3028, and 3055), with a biofilm matrix dependent on Psl. This class is defined as producing large amounts of Psl (≥20 µg/ml) and a significant reduction of biofilm biomass with PslG treatment. The prototype for class II strains is PAO1, and its derivative is PDO300. Five of the mucoid clinical isolates appear to be class III, or EPS redundant (isolates 2966, 3011, 3017, 3018, and 3064). This class is defined as producing intermediate amounts of Psl (5 to 20 µg/ml) with incomplete biofilm dissolution by PslG treatment. We presume that the residual biofilm after PslG treatment is due to Pel serving a redundant role in the matrix, as has been previously reported in nonmucoid biofilms (29). The remaining 12 strains produce very small amounts of Psl (≤5 µg/ml) and form poor biofilms. The importance of these results is that 12 out of 24 mucoid clinical isolates rely on Psl as a structural component in their biofilm matrix. Although not exhaustive, these data suggest that 50% of chronic mucoid clinical isolates from CF patients could be susceptible to treatments targeted at Psl, such as the PslG glycosyl hydrolase (43, 44) or anti-Psl antibodies (33). Further trials and testing should be conducted to apply these treatments to chronic pulmonary infections.

Prior to this study, the investigation into the contribution of Psl to mucoid strains has been minimal; however, we have identified complex and nuanced roles for Psl during chronic pulmonary infections with mucoid strains. Many of the Psl-dependent phenotypes initially observed in strain PAO1 carry into its mucoid derivative, including biofilm structure, epithelium attachment, and serum resistance. Therefore, it was surprising to us that the Psl-producing PDO300 strain colonized the murine lung poorly compared to the Psl-deficient PDO310 strain. We identified an enhanced proinflammatory response to the Psl-producing strain, which is most likely responsible for the improved clearance of this strain. Development of an improved chronic pulmonary infection model would allow investigation of the kinetics of bacterial load and immune response as the infection progresses from acute to chronic. Studying the various phases of disease may elucidate different requirements and roles for Psl and alginate. Additionally, the efficacy of treatments targeting Psl may depend on the kinetics of administration.

## MATERIALS AND METHODS

### Bacterial strains and growth conditions.

The bacterial strains used along with genotypes are provided in Table S1 in the supplemental material. *P. aeruginosa* strains were inoculated in LBNS (10 g liter^−1^ tryptone, 5 g liter^−1^ yeast extract [pH 7.5]) at 37°C for overnight cultures in a roller unless otherwise noted. Strains were grown at 37°C on LANS (LBNS with 1.5% agar) or *Pseudomonas* isolation agar (PIA) (Difco, Detroit, MI) plates. *Escherichia coli* was routinely cultured at 37°C in lysogeny broth (LB) (10 g liter^−1^ tryptone, 5 g liter^−1^ yeast extract, 5 g liter^−1^ NaCl). Semisolid medium was prepared by adding 1.5% Bacto agar to LB. Antibiotics were added to maintain or select for plasmids in *P. aeruginosa* as follows: gentamicin (Gm) at 100 μg/ml and carbenicillin (Cb) at 300 μg/ml. Antibiotics were added to maintain or select for plasmids in *E. coli* as follows: gentamicin at 10 μg/ml and ampicillin (Ap) at 100 μg/ml.

### Flow cell biofilm study.

Inoculation of flow cells was done by normalizing overnight cultures to an optical density at 600 nm (OD_600_) of 0.05 and injecting into an Ibidi μ-Slide VI^0.4^ (catalog no. 80601; Ibidi). To seed the flow cell surface, the medium flow was suspended, and the bacteria were allowed to adhere at room temperature for 1 h. Flow of 5% (vol/vol) LBNS with 0.1% arabinose was initiated at a rate of 0.15 ml/min and continued for 48 h. Following the biofilm growth period, the flow was terminated, and the biofilms were fixed with 4% paraformaldehyde. Psl was stained with a cocktail of three monoclonal antibodies provided by MedImmune (33). The antibodies were directly labeled with Alexa Fluor 647 using the Alexa labeling kit (Life Technologies). Confocal images were obtained on a Nikon A1R live-cell imaging confocal microscope. Images were obtained with a 20× oil immersion objective. Images were processed using the FIJI software (45). Quantitative analyses were performed using the COMSTAT2 software package (46). Total biomass was determined from Z-stack images using the BIOMASS command with the threshold set at 25. Three independent biofilms were imaged and analyzed. Statistical significance was determined by a one-way analysis of variance (ANOVA) with Dunnett’s multiple-comparison test (**, *P* ≤ 0.01; ***, *P* ≤ 0.001).

### Peg biofilm assay.

Peg biofilm assays are used to determine initial attachment and biofilm formation as previously described (47). Briefly, 100 µl of log-phase culture (OD_600_ of 0.5) was inoculated into MBEC biofilm inoculator plates (Innovotech) and allowed to form biofilms for 4 h at 37°C. Biomass on the peg lid was stained for 30 min in 0.1% crystal violet, followed by three washes in water. Crystal violet was extracted from the biofilm with 100% ethanol, and the OD_595_ was determined with a SpectraMax i3 plate reader (Molecular Devices). Three replicates were performed in triplicate. Statistical significance was determined by a one-way ANOVA. followed by Dunnett’s multiple-comparison test (*, *P* ≤ 0.05; **, *P* ≤ 0.01; ***, *P* ≤ 0.001).

### PslG biofilm treatment.

Peg biofilms were formed as described above. After the 4-h incubation, plate lids with attached biofilms were transferred to fresh plates containing 150 µl of phosphate-buffered saline (PBS) with or without 86 nM purified PslG (43). Samples were incubated for 1 h at 37°C and washed three times with water. Biomass remaining on the peg lid was stained for 30 min in 0.1% crystal violet, followed by three washes in water. Crystal violet was extracted from the biofilm with 100% ethanol, and OD_595_ was determined with a SpectraMax i3 plate reader (Molecular Devices). Five replicates were performed in triplicate. Statistical significance was determined by a one-way ANOVA, followed by Bonferroni’s posthoc test (*, *P* ≤ 0.05; **, *P* ≤ 0.01; ***, *P* ≤ 0.001).

### Epithelial cell attachment assay.

A549 human lung epithelial cells were grown in Dulbecco modified Eagle medium (DMEM) to approximately 90% confluence in 24-well plates. Bacterial cells were added at an MOI of 10, and the plates were centrifuged for 5 min at 300 × *g*. Infections were incubated for 1 h at 37°C with 5% CO_2_. The cultures were washed five times with PBS and then incubated for 10 min with 1 ml of PBS containing 1% saponin in each well to lyse the epithelial cells. Serial dilutions of the lysates were performed to determine the number of adherent *P. aeruginosa* cells. Data are reported as a percentage of adherent cells of the infectious dose. Experiments were performed twice in triplicate. Statistical significance was determined by a one-way ANOVA, followed by Dunnett’s multiple-comparison test (***, *P* ≤ 0.001). Each mutant was compared to its respective mucoid or nonmucoid parental strain.

### Lactate dehydrogenase assay.

Release of lactate dehydrogenase was measured using the CytoTox 96 nonradioactive cytotoxicity assay kit (Promega). A549 human lung epithelial cells were grown in DMEM to approximately 90% confluence in 96-well plates. Bacterial cells were added at an MOI of 10, and the plates were centrifuged for 5 min at 300 × *g*. Infections were incubated for 16 h at 37°C with 5% CO_2_. Ten microliters of lysis solution was added to the maximum LDH release control samples 45 min prior to harvest. Release of lactate dehydrogenase was measured using the CytoTox 96 nonradioactive cytotoxicity assay kit (Promega). Samples were centrifuged at 300 × *g* for 5 min, and 50 µl of supernatant was transferred to a new 96-well plate. Fifty microliters of CytoTox 96 reagent was added to each well and incubated for 30 min at room temperature. Stop solution was added, and the OD_490_ was recorded. The average OD measurement for the medium-only control was subtracted from each value to normalize for background. The percent cytotoxicity was calculated by dividing the LDH release from the experimental sample by the average LDH release from the maximum LDH release control samples. Five independent experiments were performed in triplicate. Statistical significance was determined by a one-way ANOVA, followed by Dunnett’s multiple-comparison test (***, *P* ≤ 0.001). Each mutant was compared to the mucoid parental strain.

### Opsonization assay.

Opsonization of bacteria was determined as previously described (21). Briefly, cells were pelleted from 1 ml of log-phase culture (OD_600_ of 0.5) and resuspended in 500 µl of 20% pooled normal human serum (Complement Technologies) and incubated at 37°C for 5 min. Following three washes with PBS, samples were blocked with PBS containing 1% bovine serum albumin (BSA) for 20 min and stained with anti-human C3 (Complement Technologies) and Alexa Fluor 647 secondary antibodies (Life Technologies). Samples were analyzed in triplicate (30,000 events each) on a BD FACSCanto II flow cytometer. For analysis, gates were set on unstained samples so that ~0.5% of the population was positive for allophycocyanin (APC) fluorescence. Statistical significance was determined by one-way ANOVA, followed by Dunnett’s multiple-comparison test (***, *P* ≤ 0.001).

### Serum sensitivity assay.

Bacterial killing by serum was determined as previously described (21). Briefly, cells were pelleted from 1 ml of log-phase culture (OD_600_ of 0.5), resuspended in 500 µl of 50% pooled normal human serum (Complement Technologies), and incubated at 37°C for 20 min. The reaction was stopped by the addition of EDTA to 10 mM, and samples were diluted and plated for colony enumeration. Data are reported as the percentage of serum-treated bacteria recovered compared to PBS-treated samples. Three experiments were performed in triplicate, and statistical significance was determined by one-way ANOVA, followed by Dunnett’s multiple-comparison test (**, *P* ≤ 0.01).

### Murine infections.

Six-week-old female BALB/c mice (Jackson Laboratory) were acclimated for 5 to 7 days prior to infection. The intranasal infection was performed as previously described (48). Briefly, mice were lightly sedated with isoflurane (Butler) and intranasally inoculated with 30 µl of PBS containing 10^8^ bacteria. The animals were sacrificed, and the lungs were aseptically harvested and homogenized in sterile PBS. Homogenates were serially diluted in PBS and plated on PIA for total bacterial load. Statistics were performed using an unpaired two-tailed Student’s *t* test. Three mice were infected with each strain of bacteria in three independent experiments. All animal procedures were conducted in accordance with Ohio State University IACUC protocol 2009A0177-R1.

### Cytokine analysis.

IL-6 was quantified in lung homogenate using the OptEIA mouse IL-6 ELISA set (BD) following the manufacturer’s directions. Briefly, homogenates were diluted 1:25 in assay diluent prior to analysis. Homogenates from three mice were assayed in triplicate for each strain infection. Statistical significance was determined by one-way ANOVA, followed by Bonferroni’s posthoc test (***, *P* ≤ 0.001).

### Isolation of mucoid clinical isolates from sputum.

Deidentified clinical isolates from CF sputum used in this study are housed in the Wozniak Strain Database and are indicated by their strain number. These samples were obtained under a material transfer agreement with the Cystic Fibrosis Center and Laboratory Services at Nationwide Children’s Hospital in Columbus, Ohio. The mucoid phenotype was confirmed by plating on PIA.

### Quantification of Psl.

Psl immunoblotting and quantification were performed as previously described (32). Psl was extracted from 1.0 ml of a culture with an OD_600_ of 1.0 to normalize for cell number. Results are reported as micrograms of Psl per culture with an OD_600_ of 1.0. Psl was extracted from the cell pellet in boiling 0.5 M EDTA, followed by treatment with proteinase K. Samples were diluted 1:10 in Tris-buffered saline with Tween 20 (TBST) (20 mM Tris, 137 mM NaCl, 0.1% Tween 20 [pH 7.6]) and spotted onto nitrocellulose for immunoblotting. Immunoblots were blocked in 10% skim milk and then incubated with Psl-specific rabbit antibody (MedImmune). After the immunoblots were washed, the secondary antibody (donkey anti-rabbit; GE Healthcare) was added at a 1:10,000 dilution. After a last wash, the blot was treated with SuperSignal West Dura extended-duration substrate per the manufacturer’s instructions (Pierce). Three biological replicates were performed in triplicate. Blots were visualized using the Bio-Rad Chemidoc system. Densitometry was performed with the Quantity One software (Bio-Rad) and compared to a standard curve of pure Psl (49).

### Carbazole assay.

Alginate quantification was performed as previously described (32). Bacterial cultures were scraped from PIA and resuspended in PBS, and bacteria were collected from 2 ml of 1.0 OD_600_ by centrifugation. Alginate was precipitated from supernatants by treatment with 2% cetyl pyridinium chloride. Alginate was resuspended in 1 M NaCl, precipitated with cold isopropanol, and resuspended in saline. Alginate quantification was determined by the carbazole assay (50, 51). Alginate was treated with borate-sulfuric acid reagent (10 mM H_3_BO_3_ in concentrated H_2_SO_4_) and heated to 100°C for 15 min. Carbazole (0.1%) was added and heated to 100°C for 15 min. Absorbance was recorded at 550 nm, and concentration was determined based on a standard curve of seaweed alginate. Three independent preparations were assayed in triplicate.

10.1128/mBio.00864-17.1 TEXT S1 References for Table S1. Download TEXT S1, DOCX file, 0.1 MB. Copyright © 2017 Jones and Wozniak. 2017 Jones and Wozniak This content is distributed under the terms of the Creative Commons Attribution 4.0 International license.
