# Supplementary figures and images for: Psl Produced by Mucoid Pseudomonas aeruginosa Contributes to the Establishment of Biofilms and Immune Evasion
Source: mBio. 2017 Jun 20;8(3):e00864-17. doi: 10.1128/mBio.00864-17 (PMC5478896; doi:10.1128/mBio.00864-17)

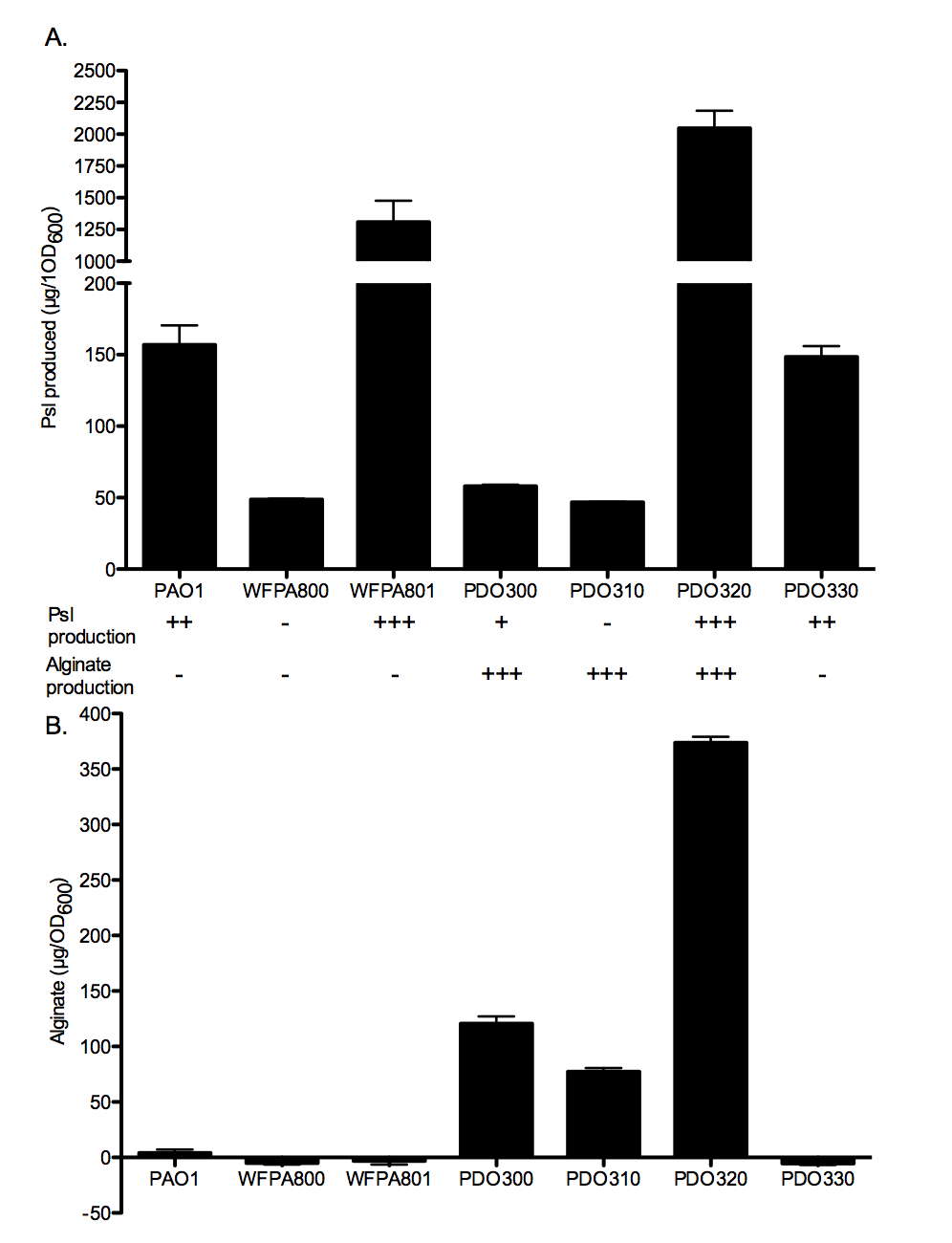

Supplement: FIG S1 [file mbo003173355sf1.tif]

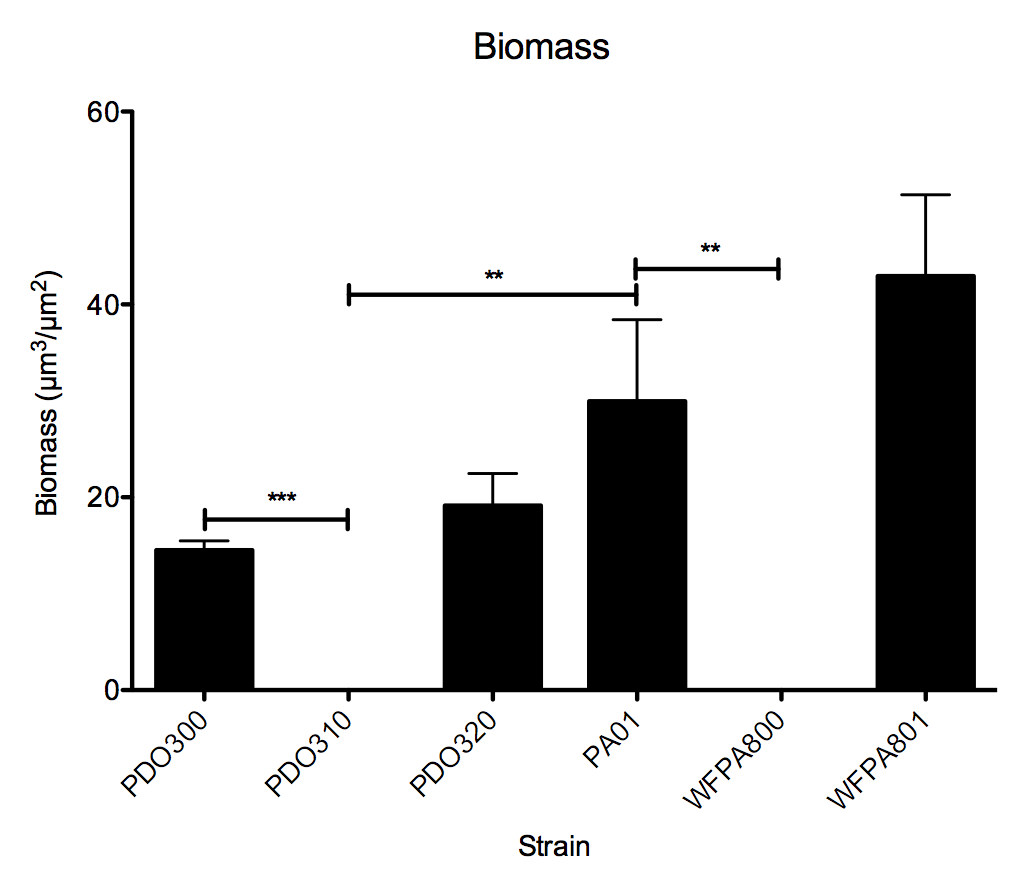

Supplement: FIG S2 [file mbo003173355sf2.tif]
