# Supplementary material for: Psl Produced by Mucoid Pseudomonas aeruginosa Contributes to the Establishment of Biofilms and Immune Evasion
Source: mBio. 2017 Jun 20;8(3):e00864-17. doi: 10.1128/mBio.00864-17 (PMC5478896; doi:10.1128/mBio.00864-17)
Supplement: TABLE S1 [file mbo003173355st1.docx]

**Table S1. Strains used in this study.**

| Strain | | Relevant genotype | Source |
| --- | --- | --- | --- |
| *P. aeruginosa* strains | | | |
|  | PAO1 | Nonmucoid parental strain | (1) |
|  | WFPA800 | PAO1 *pslA* operon promoter deletion, nonmucoid | (2) |
|  | WFPA801 | PAO1 *psl-araC-pBAD* promoter replacement, nonmucoid | (2) |
|  | PDO300 | PAO1 *mucA22,* mucoid | (3) |
|  | PDO310 | PAO1 *mucA22 pslA* operon promoter deletion*,* mucoid | (4) |
|  | PDO320 | PAO1 *mucA22 psl-araC-pBAD* promoter replacement*,* mucoid | (4) |
|  | PDO330 | PAO1 *mucA22 algD* operon promoter deletion*,* nonmucoid | This study |
|  | NCH Clinical Isolates | De-identified sputum isolates obtained from Nationwide Children’s Hospital | This study |
