# Supplementary material for: Psl Produced by Mucoid Pseudomonas aeruginosa Contributes to the Establishment of Biofilms and Immune Evasion
Source: mBio. 2017 Jun 20;8(3):e00864-17. doi: 10.1128/mBio.00864-17 (PMC5478896; doi:10.1128/mBio.00864-17)
Supplement: TEXT S1 [file mbo003173355s1.docx]

**References for Table S1**

1. Holloway BW. 1955. Genetic recombination in *Pseudomonas aeruginosa*. Journal of General Microbiology 13:572–581.

2. Ma L, Jackson KD, Landry RM, Parsek MR, Wozniak DJ. 2006. Analysis of *Pseudomonas aeruginosa* conditional *psl* variants reveals roles for the Psl polysaccharide in adhesion and maintaining biofilm structure postattachment. J Bacteriol 188:8213–8221.

3. Mathee K, Ciofu O, Sternberg C, Lindum PW, Campbell JI, Jensen P, Johnsen AH, Givskov M, Ohman DE, Molin S, Høiby N, Kharazmi A. 1999. Mucoid conversion of *Pseudomonas aeruginosa* by hydrogen peroxide: a mechanism for virulence activation in the cystic fibrosis lung. Microbiology 145 ( Pt 6):1349–1357.

4. Ma L, Wang J, Wang S, Anderson EM, Lam JS, Parsek MR, Wozniak DJ. 2012. Synthesis of multiple *Pseudomonas aeruginosa* biofilm matrix exopolysaccharides is post-transcriptionally regulated. Environ Microbiol 14:1995–2005.
